# Cognitive Underpinnings of Functional Reading Difficulties in Polish Adults

**DOI:** 10.3390/brainsci16050438

**Published:** 2026-04-22

**Authors:** Katarzyna Chyl-Tanaś, Marcin Szczerbiński, Artur Pokropek

**Affiliations:** 1 Educational Research Institute, 01-180 Warsaw, Poland; a.pokropek@ibe.edu.pl; 2 School of Applied Psychology, University College Cork, T23 TK30 Cork, Ireland; m.szczerbinski@ucc.ie; 3 Institute of Philosophy and Sociology, Polish Academy of Sciences, 00-330 Warsaw, Poland

**Keywords:** functional illiteracy, adult literacy, reading comprehension, low literacy, cognitive profiles, Simple View of Reading

## Abstract

**Highlights:**

**What are the main findings?**

• Good readers understood texts based on how well they understood spoken language, but struggling readers were held back by reading words too slowly and with too much effort.
• Adults who struggle with reading are not all the same. In our study, they fell into three groups: most had trouble with general language skills (54%), many had typical cognitive reading abilities (38%), and a small number had severe, dyslexia-like problems with reading words (9%).

**What are the implications of the main findings?**

• Adult literacy instruction should be flexible and target the skills learners actually need: vocabulary and reading strategies for those with oral language difficulties, and decoding for those who struggle with word reading.
• Because many adults with low reading scores had typical cognitive skills, support programs also need to address outside barriers like low motivation, learned helplessness, and limited technology access and reading habits.

**Abstract:**

**Background/Objectives**. Low functional literacy in adulthood is a growing issue, yet the cognitive profiles of struggling adult readers in transparent orthographies remain under-researched. This study investigated the cognitive and reading-related predictors of reading comprehension in Polish adults using the Simple View of Reading framework. **Methods**. We analyzed data from 158 adults recruited as typical readers (TRs, n = 76) and low readers (LRs, n = 82) based on functional reading comprehension scores. Participants completed comprehensive behavioral assessments measuring decoding, listening comprehension, phonological awareness and memory (PA&M), rapid automatized naming (RAN), and language abilities. **Results**. Path analysis indicated that both decoding and listening comprehension independently and significantly distinguished TRs from LRs (McKelvey-Zavoina R^2^ = 43.6%). However, multigroup analysis revealed differing mechanisms between the two groups: reading comprehension in TRs was driven primarily by listening comprehension, whereas comprehension in LRs was constrained by decoding, which was heavily influenced by RAN and PA. Furthermore, latent profile analysis uncovered significant heterogeneity among the struggling readers, identifying three distinct subgroups: a language-deficit profile (53.8%), a cognitively typical profile (37.5%), and a dyslexic profile (8.8%). These distinct subprofiles aligned with varied background factors, including self-reported dyslexia and early home literacy environment. **Conclusions**. This study demonstrates that functional reading difficulties in Polish adults are not homogeneous. The identification of three distinct profiles among low readers - language deficit, cognitively typical, and dyslexic - highlights that interventions must be tailored to specific cognitive needs. These findings underscore the necessity for specialized adult-literacy support in Poland to address the growing challenge of low functional literacy and its associated social and economic risks.

## 1. Introduction

In high-income countries, low literacy in adulthood rarely takes the form of absolute illiteracy. Rather, it is a lack of *functional* literacy, which encompasses the capacity to understand, evaluate, and engage with written information to navigate modern society [1,2]. Deficits in these fundamental skills correlate with adverse life outcomes, including reduced employability and social participation [3,4]. Data from the PIAAC cycle 2 demonstrate this is a growing challenge in Poland, where 39% of the adult population performs at or below level 1 in literacy [5], a steep increase from the 18.8% recorded in cycle 1 [6]. Despite the social and economic risks associated with this decline, Poland lacks an educational policy and support programs tailored for struggling adult readers. Mapping the cognitive and reading-related deficits that constrain adult reading in Polish is the first step to developing effective interventions.

### 1.1. Simple View of Reading

The Simple View of Reading (SVR) [7,8] is the theoretical framework that has informed much of research into the mechanisms of individual differences in reading comprehension. The framework posits that reading comprehension (RC) is the product of two processes: decoding and listening comprehension. Decoding refers to the ability to recognize printed words, while listening comprehension encompasses the ability to interpret spoken language. In typical adult readers, decoding is highly mastered, and its contribution to RC variance decreases with age [9]. However, meta-analyses of adults with low literacy reveal that decoding remains a constraining predictor of their RC, manifesting as slow, effortful word recognition [10].

Importantly, poor-reading adults differ from children matched on reading levels [11]. They possess more background knowledge and utilize different reading strategies, relying less on phonological decoding and more on whole-word recognition and prior context than children do [12]. Although factors like vocabulary and background knowledge also contribute to children’s reading, extended variants of the SVR highlight how their relative importance shifts to define the distinct adult profile, shaped by decades of accumulated life experience and varied print exposure. For example, Braze and colleagues [13] introduced vocabulary as a separate third predictor, arguing it exerts a direct effect on adult RC over and above decoding. Snow [14] argues that skills in academic language, perspective-taking, and argumentation are additional important predictors of RC. Background knowledge was also shown as a unique, direct predictor of RC for struggling adults, and suggested as an extension to the model [15].

Extended variants of SVR share one limitation: just like the original SVR, they were developed mostly in the context of English. English has an opaque orthography where irregular letter–sound mappings make decoding accuracy a persistent hurdle [16]. In languages using more transparent orthographies, the profile of the struggling adult reader may shift.

### 1.2. Cognitive Predictors of RC in Transparent Orthographies

*Decoding.* Orthographic transparency exists on a continuum. While Polish is much more transparent than English, it is not perfectly shallow like Finnish or Italian. Nevertheless, its highly consistent feedforward (grapheme-to-phoneme mappings) for reading enable most individuals, including struggling readers, to achieve basic decoding accuracy relatively easily [16,17]. The decoding deficit therefore manifests not as an inability to read words accurately, but as a failure to achieve automatized, fluent word recognition [18]. Slow decoding perpetuates the bottleneck described by verbal efficiency theory [19]: processing resources that should serve text comprehension remain occupied at word-level decoding. Evidence from studies on adults using transparent orthographies suggests that once listening comprehension enters a regression model, the unique contribution of word reading to RC diminishes, pointing to decoding fluency as a necessary but insufficient constraint [20]. *Phonological Awareness* *and Phonological Memory* (PA&M). In transparent orthographies, PA&M’s direct contribution to reading fluency declines with schooling, as the regularity of the script allows it to be acquired relatively early [21]. Its effects on RC appear to be mediated through word-reading speed [22]. However, contrary to the assumption that PA&M deficits resolve once basic decoding is acquired, research demonstrates that PA&M remains a persistent, continuous area of difficulty for a notable subset of low-literacy adults using transparent orthographies, such as German [18] and Italian [23].

*Rapid automatized naming (RAN).* Alongside PA&M, rapid automatized naming (RAN) represents a critical foundational skill. In transparent orthographies, reading speed rather than accuracy is often considered the primary dimension distinguishing skilled from struggling adult readers [18,24]. The double-deficit hypothesis [25] proposes that phonological awareness and naming speed constitute two separable sources of reading dysfunction, with individuals exhibiting deficits in both showing the most severe impairments. RAN tasks, which require rapid serial retrieval of highly familiar visual stimuli, are thought to reflect the efficiency of forming orthographic representations, a process critical for fluent word recognition [25]. Although a meta-analysis across scripts of varying transparency found that RAN effects were larger in opaque than in transparent orthographies [26], RAN remains a robust correlate of reading fluency across all orthographic depths.

*Listening comprehension*. Longitudinal research in Finnish demonstrated that while reading fluency predicted RC in grade 1, its direct contribution disappeared by Grade 2, leaving listening comprehension as the sole stable SVR predictor of RC across subsequent years [27]. In typical adult readers of Portuguese, Listening Comprehension displayed a larger contribution to RC than decoding, with vocabulary influencing RC both directly and indirectly, via listening comprehension [28]. This pattern extends to adults with reading difficulties: young adults with poor RC in German performed significantly below age-expected norms on listening comprehension tasks [20]. However, the deficit in listening comprehension among low-literacy German adults is not a consequence of limited language exposure: Vágvölgyi and colleagues [22] showed that both oral grammatical and semantic comprehension contributed independently of listening comprehension to RC, suggesting genuine linguistic processing deficits rather than impoverished vocabulary input alone.

*Broader oral language competencies.* While decoding, PAM, and RAN constrain the mechanical efficiency of reading, higher-order semantic and structural abilities are required to construct meaning. Broader language skills support RC at multiple levels. Vocabulary facilitates lexical access and semantic inference generation; however, as noted above, its effects appear mediated through listening comprehension rather than acting directly on RC in struggling adult samples [13]. Morphological awareness—knowledge of word roots and affixes—constitutes a separable, direct predictor of adult RC above and beyond decoding [10]. Less is known about the predictive power of syntactic skills in this context [10], but it is plausible that they also contribute.

*Other cognitive and contextual factors*. Individual differences in RC are underpinned by domain-general resources, starting with general cognitive ability (IQ). Struggling adults often demonstrate typical fluid IQ, suggesting that their difficulties are not a byproduct of general reasoning deficits [18]. Instead, crystallized intelligence, representing accumulated semantic and world knowledge, serves as a core driver of comprehension. Measured through verbal reasoning and vocabulary, the effects of crystallized intelligence are significant, but largely mediated through listening comprehension [10]. Supporting this knowledge base is working memory, the “mental workspace” required to maintain and integrate information across clauses. This resource acts as a constraint in adult models, as limited capacity here creates a secondary bottleneck that prevents the reader from constructing a coherent mental model of the text. Finally, background knowledge, which functions as a single integrated construct encompassing both broad academic and general information, directly predicted reading comprehension in struggling adults even after accounting for foundational language and decoding skills [15].

### 1.3. Environmental Moderators and the “Matthew Effect”

Low-literacy home environments can constrain children’s literacy development by limiting exposure to books, shared reading, and other literacy-rich interactions. This in turn reduces print exposure. Irrespective of the home environment, a child’s unique experience also matters. The Matthew effect, originally described by Stanovich [29], refers to a within-child reciprocal cycle in which poor decoding leads to reading avoidance (and consequently reduced print exposure), which in turn slows the growth in vocabulary and background knowledge, further widening the gap with more skilled readers. Thus, depressed comprehension skills in struggling adults often reflect a lifetime of text avoidance [13,29].

Although these two processes are empirically related, they are conceptually distinct and have different implications for intervention: low-literacy home environments point to the importance of early home literacy support, whereas the Matthew effect highlights the need to increase reading engagement and practice.

These environmental factors (intertwined with genetic and neurodevelopmental vulnerability towards language and literacy difficulties) may shape long-term educational trajectories. Many low-literacy adults report highly negative early school experiences, and research estimates that 50% to 80% of adults in adult basic education programs may have an underlying, often undiagnosed, specific learning disability [30]. Because low-SES families often lack access to diagnostic testing and interventions, these hereditary bottlenecks often remain untreated. The resulting academic frustration frequently culminates in early school dropouts.

### 1.4. Heterogeneity Among Struggling Adult Readers

Because reading comprehension is the cumulative effect of diverse factors, struggling adult readers are a heterogeneous group. For instance, while low literacy is a broader term than developmental dyslexia, the boundary between them in adulthood is porous. Unresolved dyslexia—often rooted in persistent phonological and decoding deficits—frequently underlies reading difficulties in adult populations [4]. However, rather than falling into distinct categories, low readers exhibit a continuum of profiles, and an individual’s comprehension may be constrained by a complex interplay of different factors.

Attempts to map this diversity show both the utility and the instability of subtyping. For example, five distinct subtypes were found when categorizing adult readers by oral narrative skills, but four different profiles when reading, spelling, and broader oral language abilities were analyzed jointly [31,32]. Another study reported seven subtypes and grouped them into adults with primary inadequate phonemic decoding skills, adults with deficit in word level reading and fluency, and adults with adequate skills, but insufficient comprehension, suggesting diverse methods for instruction [33]. A study of readers of German (a relatively transparent language) found that some struggling adults demonstrated typical performance in decoding or phonological processing [18]. Synthesizing these findings reveals that cross-study comparisons are obscured by two main factors: differing definitions of low reading skills based on highly variable cutoffs or grade levels [2], and inconsistent operationalizations of core components like decoding [9]. To overcome the limitations of these arbitrary definitions and operationalizations, statistical approaches like latent profile analysis (LPA) offer a robust alternative. Unlike traditional subtyping, LPA is a data-driven method that identifies subpopulations based on continuous patterns of performance across different domains. By applying LPA to adult reading data, researchers can determine whether the variance among struggling readers clusters into distinct profiles without imposing artificial thresholds.

### 1.5. The Current Study and Research Questions

While extended SVR models provide a framework for explaining individual differences in adult reading comprehension, data from transparent orthographies remain scarce. This study fills a critical gap in the literature by combining the SVR framework with a data-driven latent profile analysis (LPA) approach to map adult-specific reading profiles in a transparent orthography. Utilizing path analysis, we first tested the structural predictive pathways of adult comprehension, using our functional reading measure (*Czytest*) both for grouping and as the continuous outcome variable. Then, by applying LPA, we mapped the subpopulations of struggling readers without imposing artificial thresholds. To this end, we asked four research questions (RQs):

**RQ1.**  *Do cognitive and reading-related variables distinguish typical from low reading comprehenders in a Polish sample?*

We expected that both major components—decoding and listening comprehension—would directly predict whether an adult is classified as a typical or poor reader. Furthermore, we expected foundational skills, particularly phonological awareness and rapid naming speed, to exert strong indirect effects that cascade through decoding, and language skills through listening comprehension, to influence overall reading status [34].

**RQ2.**  *Does the mechanism driving RC differ between groups? Specifically, does the predictive weight of decoding versus listening comprehension change depending on a reader’s proficiency level?*

We predicted that the relative contribution of different skills towards explaining variability in RC would differ across the groups. For typical readers whose decoding has become fully automatized, RC variance would be driven mostly by listening comprehension. In poor readers, we expected that both decoding and listening comprehension would predict functional RC scores.

**RQ3.**  *Is the low reading comprehension group uniform, or can qualitatively distinct subprofiles be identified through latent class analysis?*

Given the cumulative and diverse nature of adult reading difficulties, we predicted that the poor-reader group would be heterogeneous. Using LPA, we expected to identify qualitatively distinct subprofiles. Since different typologies are reported in the literature, depending on the type of analysis and introduced variables [2], the typology of these profiles is yet to be determined. Nonetheless, we expected to see a profile of poor decoders—adults with severe dyslexia.

**RQ4.**  *If subprofiles are found, will we find differences in their developmental history and environmental backgrounds, such as early print exposure, education level, or self-reported learning difficulties?*

This analysis will be exploratory, driven by the RQ3 findings. However, we hypothesized that subprofiles, if found, would align with distinct environmental and educational histories reported in the questionnaires. Individuals fitting the “dyslexic” profile should report significantly higher rates of dyslexia diagnoses and early school struggles.

## 2. Materials and Methods

### 2.1. Recruitment and Participants

Participants were recruited between June and October 2024 via job-portal advertisements, social media, snowball referrals, and partnerships with social welfare centers. Of 1706 individuals who expressed interest online, 538 met the educational eligibility criteria (maximum secondary education; active university students excluded) and received a personalized link to the reading comprehension online screening measure [35]. Of the 359 individuals who attempted the test, 307 completed it, and 189 of these were subsequently invited to the full study on the basis of their reading comprehension score, of whom 163 completed assessment sessions. The participants were reimbursed for both stages of the study (reading comprehension test—PLN 50, assessment session—PLN 100). All participants electronically signed an informed consent form. Both informed consent and all information about the study were written with maximum clarity according to plain-language standards [36]. The study was approved by the Ethics Committee of the Educational Research Institute, Warsaw, Poland (approval date 25 October 2023), and was conducted in accordance with the American Psychological Association’s Ethical Principles of Psychologists and Code of Conduct.

Participants were classified into two groups based on their score on Czytest [35]. Those scoring ≥31 points were assigned to the typical readers group (TRs; n = 76, 48.1%), and those scoring ≤25 points were assigned to the low readers group (LRs; n = 82, 51.9%). Individuals scoring in the intermediate range (26–30 points; n = 5) were excluded at the recruitment stage to increase the discriminative clarity of the classification. This approach is analogous to the extreme-group design commonly employed in reading research [37], and ensures that the two groups are clearly differentiated on functional reading ability. We note that this intentional gap precludes interpretation of continuous Czytest scores in the analytic sample. By removing the intermediate range, this design enhances the contrast between distinct proficiency levels, allowing the multigroup path analysis to isolate mechanisms that differentiate reading failure from reading success. However, as discussed in Section 4.8, this truncation reduces within-group variance and may inflate between-group effect sizes. The psychometric development of Czytest, including the derivation of score benchmarks aligned with PIAAC literacy levels, is described in detail in a separate article [35].

The final sample comprised 158 adults (M age = 41), after excluding four initial pilot participants and one person who later reported holding a university degree. Three remaining participants still pursuing education (2 LRs, 1 TRs) were retained, as their small number did not warrant separate treatment. Participants were recruited across different types of settlements and geographic locations within Poland and covered a range of education levels, with the intentional exclusion of tertiary-educated adults, since the likelihood of finding low-literacy individuals among them is smaller [38]. For their demographic characteristics, see Table 1.

To verify that the two groups were comparable on demographic characteristics, we tested group differences using independent-sample *t*-tests for age and chi-squared tests for categorical variables. Age did not differ significantly between the groups: TRs (*M* = 40.24, *SD* = 11.91) vs. LRs (*M* = 41.76, *SD* = 14.25), *t*(154.4) = −0.73, *p* = 0.467. Sex distribution was comparable across groups (TRs: 53.9% women; LRs: 63.4% women), χ^2^(1) = 1.10, *p* = 0.295. Place of residence did not differ significantly between groups, χ^2^(3) = 5.49, *p* = 0.139. Educational attainment differed significantly between groups, χ^2^(2) = 13.63, *p* = 0.001. The TR group had a much higher proportion of participants with general/technical secondary education (40.8% vs. 14.6%), while the LR group had a higher proportion of participants with vocational secondary education (65.9% vs. 46.1%).

It is important to emphasize that this sample is not representative of the general adult Polish population, which includes a substantial proportion of tertiary-educated individuals [39]. By excluding university graduates, our sample’s educational composition aimed to oversample adults at risk of low reading skills [5]. Consequently, this design precludes generalizing these results to population level.

### 2.2. Materials

**Figure 1:**
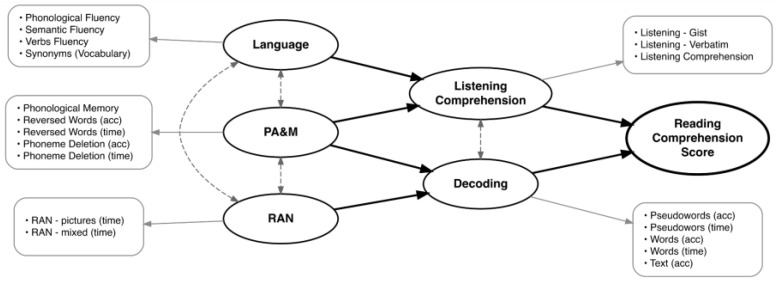
Theoretical structural model of reading comprehension. Thick solid black arrows represent the structural paths for the main theoretical hypotheses. Dashed, double-headed arrows indicate covariances between variables. Thin gray arrows represent the measurement model, linking latent constructs to their observable indicators.

The first stage after recruitment was the completion of an online, self-administered reading comprehension test. Then, all chosen participants completed a battery of tasks administered by a trained researcher in a one-hour individual online session via Google Meet. Standardized normed tests were used where available. Given the scarcity of such data for Polish adults, some tasks were adapted from literacy assessment batteries originally designed for children, or they were developed specifically for the purpose of this study. The theoretical structural model of reading comprehension together with all measured variables is presented in Figure 1.

*Reading Comprehension.* Functional reading comprehension was assessed using an online self-administered test developed and validated for use with Polish-speaking adults. Czytest measures reading comprehension in its functional dimension: the ability to extract, integrate, and use information from everyday written texts. The test served a dual role in the present study: as a screening and classification tool (group assignment), and as a dependent variable in multigroup confirmatory analyses. Results of the normalization study confirmed the tool’s unidimensionality and high reliability (α = 0.88). For full psychometric properties of Czytest, including its derivation from PIAAC literacy benchmarks and cutoff determination, see Chyl-Tanaś et al. [35].

### 2.3. Word Recognition and Decoding

• Rapid word reading. Participants read a list of 69 real Polish words aloud as quickly as possible. Accuracy (number correct) and response time were recorded. The task was adapted from *Test Dekodowania* [40].
• Rapid pseudoword reading. The same procedure was applied to 69 pseudowords [40].
• Text reading. Participants read a short functional text (an announcement) aloud at their natural pace. They were instructed to pretend they were reading it to an elderly person interested in its content. This task was developed for the purpose of this study.

### 2.4. Listening Comprehension

• Listening comprehension with explicit recall questions. Following a weather forecast recording, participants answered factual questions. This task was adapted from *Test Kompetencji Komunikacyjnej* [41].
• Listening comprehension tasks with free recall. Participants listened to short authentic-style recordings (telephone message, supermarket announcement, local celebration announcement, local radio news, advertisement, and podcast about mortgages) and freely recalled all information they remembered. Responses were recorded, transcribed, and later coded by a trained researcher using a predefined scoring rubric to ensure consistency. Two measures served as a score from this task: (1) gist units—narrative and descriptive information units, capturing the overall meaning of the message, consistent with fuzzy-trace theory [42], and (2) verbatim units—specific operational data points critical to correctly retrieve the main message. This task was developed in-house (alpha = 0.86).

### 2.5. Phonological Awareness and Memory (PA&M)

• Phoneme deletion. Participants removed a specified phoneme from 6 spoken words. Accuracy and total time were recorded and served as a measure of this task. The task was adapted from *Test Dekodowania* [40].
• Reverse words. Participants were asked to reverse 6 spoken words phoneme by phoneme (e.g., *brama*—*amarb*). Accuracy and total time were recorded and served as a measure of this task. This task was developed in-house.
• Phonological memory. Participants immediately recalled lists of 3–6 pseudowords. The score was the total of pseudowords correctly recalled. The task was adapted from *Bateria 16+* [43].

### 2.6. Rapid Automatized Naming (RAN)

• Rapid automatized naming of pictures. Participants named a series of pictures as rapidly and accurately as possible. The researcher continuously monitored their performance. If a participant skipped a line, they were instructed to immediately return to it. Minor misnaming errors and omissions were ignored, as they are rare in adults and the primary dependent variable was total naming speed, not accuracy. This version of RAN was developed in-house.
• Rapid automatized naming of pictures, letters, and digits. Participants named a series of pictures, letters, and digits, with the same scoring protocol. This version of RAN was developed in-house.

### 2.7. Language

• Verbal fluency. This task was adapted from *Test Fluencji Słownej* [44] and required participants to produce as many words as possible in 60 s in five separate trials under three different conditions:
• Phonological fluency (letters K and F).
• Semantic fluency (animals, fruits).
• Verb fluency (“What can people do?”).
• Synonym recognition. Participants selected the synonym of 10 low-frequency Polish words from a multiple-choice list. This task was adapted from the *Pathfinder General Cognitive Ability Test* [45].

*Questionnaires.* Participants completed three self-report questionnaires as part of the first (online) stage of the study.

• Background and Demographics. A demographic questionnaire collected basic sociodemographic information: sex, year of birth, native language, place of residence, educational attainment, employment status, and current enrollment in formal education. It also included items on psychophysical state at the time of testing: self-rated health, vision, and sleep. The remaining items—covering subjective financial situation, mother’s education, frequency of digital device use, numeracy use in daily life, and (for employed participants) reading frequency and task autonomy at work—were drawn from the PIAAC Background Questionnaire [46]
• Reading History and Habits Questionnaire. Three items on retrospective reading difficulty in primary school (ease of learning to read, reading skill relative to peers, spelling difficulties) were adapted from the Polish version of the Adult Reading History Questionnaire (ARHQ; [47]). One item (the frequency of needing help when reading medical instructions or leaflets) was drawn from the Single-Item Literacy Screener (SILS; [48]). Reading habits (frequency of book reading; frequency of reading other texts such as news or practical online information), childhood home literacy environment (number of books at home at age 14), and social attitude items (interpersonal trust, external locus of control) were adapted from the PIAAC Background Questionnaire [46]. Finally, the participants were asked whether they had been diagnosed with dyslexia and about the history of reading difficulties in their immediate family.
• Test Engagement Questionnaire. Immediately following completion of Czytest, participants completed a 3-item measure of test engagement. Items assessed estimated performance, concentration, effort invested, and time pressure during the test, as well as the type of device and input method used. This questionnaire was designed to flag low-effort responses and to capture potential confounds related to device type.

All analyses were conducted in R (version 4.5.2) with package versions managed via renv to ensure reproducibility. All data, a full list of package versions and analysis scripts are available at the project's OSF page https://osf.io/hf86a accessed on 16 April 2026).

Response time variables with skewness exceeding |1| were log-transformed prior to analysis. The sign was subsequently reversed for all time-based indicators so that higher values consistently reflected better performance. Item-level responses were aggregated into composite scores (sum scores for accuracy measures, mean scores for normalized fluency indices), which were then z-scored using the full-sample mean and SD.

Unit-weighted composites, computed as the mean of standardized indicators within each construct, served as manifest predictors in the structural model. This approach was chosen given the small per-group samples (n ≈ 80). Although unit-weighted composites do not account for measurement error, they have been shown to perform comparably to factor scores under conditions of homogeneous loadings [49]. We acknowledge this as a limitation where loadings diverge. Nonetheless, internal consistency of the composites was acceptable to excellent across all five constructs—PA&M (α = 0.74), language (α = 0.75), listening comprehension (α = 0.78), decoding (α = 0.89), and RAN (α = 0.93)—with mean inter-item correlations ranging from 0.36 to 0.86. Moreover, unit-weighted composites are more stable and comparable across samples than factor scores, as their weights carry zero sampling variability [50]. This property is especially advantageous in small-sample multigroup designs, where factor-score weights would differ between groups and between samples, introducing instability. Formal tests of measurement invariance for the CFA models did not achieve configural equivalence between groups, further supporting the decision to use unit-weighted composites, which ensure compositional invariance by construction [51].

Cronbach’s α was computed separately for LRs and TRs. Reliability was highly comparable for PA&M (α = 0.67 vs. 0.68), RAN (α = 0.92 vs. 0.92), and language (α = 0.68 vs. 0.71). Modest differences were observed for listening comprehension (α = 0.77 vs. 0.69) and decoding (α = 0.89 vs. 0.76). This probably reflects restriction of range on accuracy-based indicators in the higher-performing TR group—a pattern corroborated by Levene’s tests for several decoding and PA&M items. Since composites are unit-weighted, compositional invariance holds by construction. Differential reliability in decoding may have modestly attenuated path coefficients in the TR group and is noted as a limitation.

Path models were estimated using *lavaan* [52], with standard errors calculated via a bootstrapping procedure using 2000 iterations. Only one participant had missing data on a model variable (RAN composite, LR group). Missing data were handled via full information maximum likelihood (FIML) in the MLR model. The DWLS model predicting LR/TR group membership was estimated on the listwise-complete subsample (N = 157), as FIML is not available with the DWLS estimator in *lavaan*. LPA was conducted using the *tidyLPA* package (V 0.6-21).

## 3. Results

### 3.1. Predicting Group Membership

As a preliminary step, we evaluated baseline group differences in decoding and listening comprehension. TRs scored significantly higher than LRs on both decoding, *t*(115.91) = 4.61, *p* < 0.001, d = 0.72, and listening comprehension, *t*(155.89) = 5.87, *p* < 0.001, d = 0.93. For details, see Table 2.

To identify how these and other underlying components distinguished TRs from LRs (our first research question), we conducted a path analysis using a DWLS estimator with 2000 bootstrap iterations. Both listening comprehension (β = 0.402, *p* < 0.001) and decoding (β = 0.376, *p* < 0.01) independently predicted whether an adult was classified as a TR or LR.

Together, these variables explained 43.6% of the variance in reading status based on the McKelvey–Zavoina R^2^, which estimates the proportion of variance explained in the underlying continuous latent variable. On the observable scale, the model achieved a Nagelkerke R^2^ of 0.294 and an area under the curve of 0.783. This indicates that decoding and listening comprehension provide good discriminative ability.

**Figure 2:**
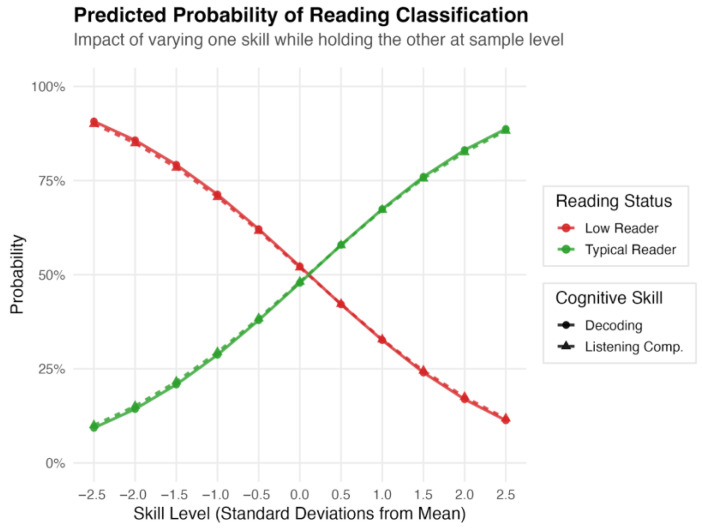
Predicted probability of low-reader classification based on decoding and listening comprehension. Notes: The curves represent the predicted marginal probabilities derived from the DWLS probit model for decoding and listening comprehension. Probabilities were calculated by varying one skill across standard deviations while holding the other constant at the mean. Solid lines represent the predicted probability trajectories for Decoding, while dashed lines represent the trajectories for Listening Comprehension. The horizontal dashed line at 50% indicates the probability threshold for classification.

Other skills influenced reading status primarily through indirect mediating pathways. PA&M had a significant total effect on reading status (β = 0.293, *p* < 0.001) mediated by both decoding (β = 0.475, *p* < 0.001) and listening comprehension (β = 0.286, *p* < 0.01). Language predicted listening comprehension (β = 0.464, *p* < 0.001), resulting in a significant indirect effect (β = 0.186, *p* < 0.01). RAN predicted decoding (β = 0.424, *p* < 0.001), translating into a significant indirect effect (β = 0.159, *p* < 0.05). Overall, the exogenous variables successfully explained 62.4% of the variance in decoding and 47.8% of the variance in listening comprehension. For predicted probabilities, see Figure 2.

To illustrate these effects within the studied demographic, we calculated predicted probabilities based on the model’s parameters. For an individual performing at the sample mean for both decoding and listening comprehension, the predicted probability of being classified as an LR is 51.6%. However, for an individual performing one standard deviation below the sample mean in decoding or listening comprehension, this predicted risk is much higher, at 71.3%, and 70.6%, respectively. Conversely, individuals performing one standard deviation above the sample mean in decoding or listening comprehension have a lower predicted risk of being an LR, at 32.6% and 32.8%, respectively.

*Robustness Check: Controlling for Testing Modality*. Because participants self-administered the screening test, preliminary analyses revealed that LRs disproportionately utilized mobile devices compared to typical readers (58.5% vs. 28.9%; see Supplement Section S1). To ensure core findings were not artifacts of this modality difference, we estimated a supplementary DWLS model including device type (0 = computer, 1 = mobile) as a direct covariate predicting group classification.

The inclusion of this covariate did not alter the primary findings. Even when controlling for testing modality, both decoding (beta = 0.369, *p* < 0.001) and listening comprehension (beta = 0.341, *p* < 0.001) remained robust, independent predictors of functional reading difficulties. Full parameter estimates for this covariate-adjusted model are provided in Supplement Section S1.

### 3.2. Mechanisms of Reading in the Two Groups

**Figure 3:**
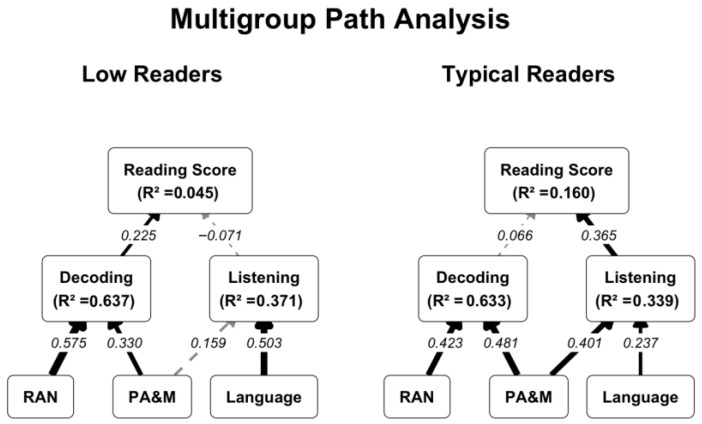
Structural pathways of reading comprehension for typical vs. low readers. Notes: R^2^ is provided within the nodes for endogenous variables. Solid black arrows indicate statistically significant pathways (*p* < 0.05). Arrow thickness is scaled to the absolute magnitude of the β coefficient. Dashed gray arrows represent non-significant pathways. Covariance pathways are omitted for visual clarity. RAN = rapid automatized naming; PA&M = phonological awareness and memory.

To determine whether the predictive mechanisms of reading comprehension differed by proficiency level (our second research question), a multigroup path analysis was estimated. The model tested the structural pathways model separately for TRs and LRs. Parameters were estimated using maximum likelihood with robust standard errors (MLRs), and significance was evaluated using 95% confidence intervals derived from 2000 bootstrap iterations (scaled χ^2^(10) = 13.713, *p* = 0.186, robust CFI = 0.992, robust TLI = 0.975, robust RMSEA = 0.062, SRMR = 0.030). To test whether the structural pathways differed between groups, this baseline model was compared against a constrained model where all regression paths were forced to be equal across both groups. A Satorra–Bentler scaled likelihood ratio test revealed that the constrained model fit the data significantly worse (Δχ^2^(6) = 46.585, *p* < 0.001), confirming that the cognitive and reading-related mechanism of reading differed between TRs and LRs (See Figure 3).

For TRs, decoding did not constrain reading comprehension (b = 0.039, 95% CI [−0.139, 0.198], β = 0.066). Instead, variance in reading comprehension was driven by listening comprehension (b = 0.138, 95% CI [0.056, 0.235], β = 0.365), which was in turn significantly predicted by language skills (b = 0.243, 95% CI [0.031, 0.427], β = 0.237) and PA&M (b = 0.499, 95% CI [0.305, 0.735], β = 0.401). For LRs, listening comprehension was not a significant predictor of reading comprehension (b = −0.048, 95% CI [−0.205, 0.137], β = −0.071). The only direct predictor in this group was decoding (b = 0.126, 95% CI [0.054, 0.273], β = 0.225), which was strongly driven by both RAN (b = 0.502, 95% CI [0.195, 0.683], β = 0.575) and PA&M (b = 0.490, 95% CI [0.264, 0.721], β = 0.330). The model explained less variance in functional reading comprehension for LRs (R^2^ = 0.045) compared to TRs (R^2^ = 0.160). The explanatory power of our multiple regression models was poor. In both groups, individual differences in functional text comprehension were driven mostly by the factors we were unable to measure. When it came to individual differences, they were accounted for exclusively by decoding in the low-reader group and exclusively by listening skills in the typical-reader group.

*Robustness Check: Controlling for Testing Modality.* To verify that the structural mechanisms between TRs and LRs were not driven by the higher prevalence of mobile device usage in the LR group, we estimated a supplementary multigroup path analysis. In this model, device type was included as a covariate predicting continuous reading comprehension scores within both groups.

Testing on a mobile device exerted a significant, independent negative effect on reading outcomes for both TRs (beta = −0.260, *p* = 0.007) and low readers (beta = −0.296, *p* = 0.003). The inclusion of this covariate increased the proportion of explained variance (R2) in reading comprehension to 22.1% for TRs and 14.0% for LRs. Controlling for this modality penalty did not alter the distinct mechanisms separating the groups: listening comprehension remained the sole significant predictor for TRs (beta = 0.325, *p* = 0.005), while decoding remained the primary constraint for low readers (beta = 0.242, *p* = 0.001). Detailed results for this covariate-adjusted multigroup model are available in Supplement Section S1.

### 3.3. Cognitive Heterogeneity of Poor Readers

To determine whether LRs constituted a uniform group (our third research question), a latent profile analysis (LPA) was conducted on the five composite scores of the LR group. Extreme outliers (|z| > 4 on any composite) were removed (n = 2), resulting in an analytic sample of N = 80. Models specifying two to five classes with equal variance and zero covariance were compared. Although the sample-size adjusted BIC (SABIC) favored a four-class solution, the Bayesian information criterion (BIC) and the analytic hierarchy process supported a three-class model. We selected the three-class model due to its superior parsimony and classification accuracy (entropy = 0.903).

**Figure 4:**
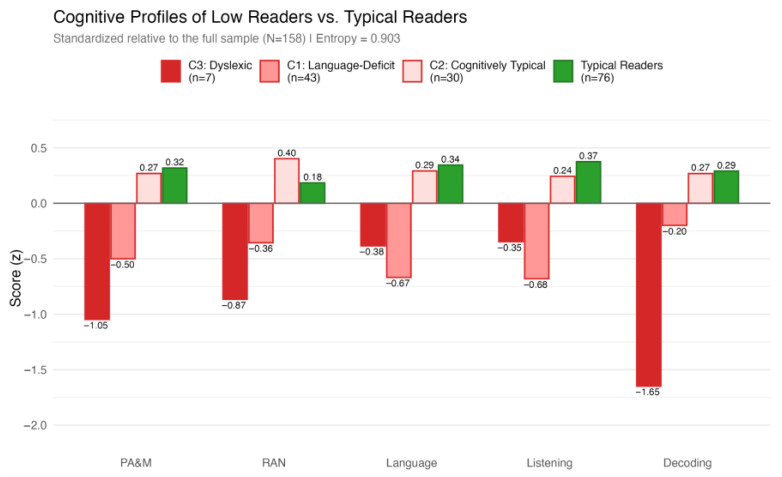
Cognitive profiles of low readers vs. typical readers. Notes: Z-scores are scaled so that negative values reflect below-average performance and positive values reflect above-average performance relative to the full sample. Latent profiles were estimated using a 3-class solution. The typical-reader group is included as a reference rather than as part of the latent profile estimation.

The LPA identified three distinct subprofiles (see Figure 4), as follows.

1. Class 1: Language Deficit (n = 43, 53.8%). This was the largest subgroup, characterized by moderate, but consistent deficits across language (z = −0.67) and listening comprehension (z = −0.68), paired with mild deficits in PA&M (−0.50) and RAN (−0.36). However, their decoding was preserved (z = −0.20).
2. Class 2: Cognitively Typical (n = 30, 37.5%). This group exhibited cognitive and reading-related performance consistently above the full-sample mean across all domains (e.g., decoding (z = 0.27), RAN (z = 0.40)), performing similarly to TRs.
3. Class 3: Dyslexic/Decoding Deficit (n = 7, 8.8%). This small, but distinct subgroup exhibited severe, pervasive deficits in decoding (z = −1.65), PA&M (z= −1.05), and RAN (z = −0.87). While their language (z = −0.38) and listening comprehension (z = −0.35) were stronger than their technical reading skills, they still fell below the full-sample mean.

We acknowledge that the sample size for LPA was modest. Still, simulation studies suggest that well-separated classes can be reliably recovered at this sample size when effect sizes between profiles are large [53], as is the case here (Cohen’s d between class 3 and class 2 exceeds 1.5 on decoding). A sensitivity analysis comparing the four-class solution yielded a similar structure, with the dyslexic class splitting into two subgroups (n = 5 and n = 9), while the language-deficit (n = 39) and cognitively typical (n = 27) profiles remained stable. Average posterior classification probabilities were 0.94, 0.92, and 0.96 for classes 1, 2, and 3, respectively, indicating high classification certainty. Nonetheless, the small size of class 3 (n = 7) warrants cautious interpretation and replication in larger samples.

### 3.4. Demographic and Background Differences

To explore demographic and background differences across the four reading profiles we identified (our fourth research question) all ordinal questionnaire variables using omnibus Kruskal–Wallis tests, applying the Benjamini–Hochberg false-discovery rate (FDR) correction across this family of tests. Furthermore, differences in the prevalence of self-reported dyslexia and family history of dyslexia were assessed using Fisher’s exact tests with Monte Carlo simulations (10,000 replicates). Finally, to ensure results were not artifacts of the testing environment, we conducted a separate set of control analyses on variables related to test engagement and device usage.

Self-reported dyslexic difficulties and formal dyslexia diagnoses differed significantly across reading profiles, Fisher’s exact test, *p* = 0.008. The dyslexic profile (C3) showed the highest rate (5/7 = 71.4%) compared to typical readers (15/76 = 19.7%), language-deficit (C1; 11/43 = 25.6%), and cognitively typical (C2; 3/30 = 10.0%) profiles. However, *family* history of dyslexia did not differ significantly across profiles, Fisher’s exact test, *p* = 0.792. Rates were similar across groups: TRs (15/76 = 19.7%), C1 (11/43 = 25.6%), C2 (5/30 = 16.7%), C3 (1/7 = 14.3%).

We then evaluated self-reported reading history, habits, and socioeconomic indicators (see Table 3 and Supplement Section S2 for the full list of 19 tests; for full questions with answer options, see Supplement Section S3). Significant differences emerged primarily between the typical readers and the low-reading group, particularly the language-deficit profile (C1). Compared to typical readers, individuals in C1 reported significantly fewer books in their childhood home, lower current frequencies of reading books and everyday texts, and lower self-assessed reading skills. Retrospectively, the C1 group also reported experiencing significantly more difficulty when learning to read and spell during childhood. Socioeconomically, this group reported a worse financial situation, less frequent online shopping, lower use of math in daily life, and less use of the internet for social contact than the typical-reader group. The cognitively typical profile (C2) exhibited intermediate scores, though they reported significantly fewer books at home and lower internet social media use than typical readers, while reporting significantly less difficulty learning to spell compared to C1. The dyslexic profile (C3) also showed statistically significant difference from typical readers in the number of books at home at 14. On other measures, it consistently showed low medians, but did not differ significantly from the other groups in post hoc testing, likely due to limited statistical power associated with its small sample.

Regarding the test-taking experience, participants differed significantly in their estimated reading comprehension test performance (H = 30.41, *p*_FDR_ < 0.001) and self-reported effort (H = 15.58, *p*_FDR_ = 0.003). Dunn’s post hoc tests with Holm correction revealed that TRs estimated they had answered more questions correctly compared to both C1 (*p* < 0.001) and C2 (*p* = 0.001). Moreover, TRs reported less effort when taking the test than participants in C1 (*p* = 0.001). Across all four reading profiles, groups reported similar levels of focus (H = 3.95, *p*_FDR_ = 0.356) and perceived time pressure (H = 1.27, *p*_FDR_ = 0.737).

The device type used for the reading comprehension test also varied systematically by reading profile (Fisher’s exact *p* < 0.001). While the majority of TRs (71.1%) completed the test on a computer (desktop, laptop), LRs relied more on mobile devices (tablet, smartphone). Importantly, device type systematically impacted test outcomes, with mobile device usage associated with an approximate 2.5-point penalty on reading comprehension scores across both reading groups. Pairwise Fisher’s tests with Holm correction indicated that mobile device usage was more prevalent in the language-deficit profile (62.8%; *p* = 0.003) and the dyslexic profile (85.7%; *p* = 0.026) compared to TRs. Input method (touchscreen vs. mouse/touchpad) mirrored device type, yielding identical statistical results, and is not reported separately.

## 4. Discussion

### 4.1. SVR and Its Limits in the LRs

Our findings confirm that the SVR provides a good structural framework for distinguishing typical from struggling Polish adult readers. Both decoding and listening comprehension independently predicted functional reading status, together accounting for 44% of the underlying variance. Furthermore, foundational cognitive abilities cascaded through the model just as our theoretical framework (informed by the Simple View of Reading) predicted: PA&M and RAN supported decoding (62.4% of variance explained), while language and PA&M supported listening comprehension (47.8% of variance explained).

However, the multigroup path analysis revealed distinct ways of deploying these skills, conditional on the reader’s proficiency. For TRs, decoding no longer constrained reading comprehension: listening comprehension drove variance entirely. This reflects an automatized reading profile where word recognition demands minimal effort. Conversely, for LRs, decoding remained the primary predictor, heavily dependent on RAN and PA&M. This is consistent with the verbal efficiency theory [19], which posits that slow and effortful word recognition exhausts the working-memory resources required for reading comprehension, preventing struggling adults from effectively leveraging their listening comprehension.

While our model successfully categorized individuals into reading-proficiency groups, our findings highlight its limitations in predicting the continuous variance of functional reading scores within those groups. The structural model explained 16% of the variance in RC for TRs, but only 4.5% for LRs. Such low predictive power can be partly explained by truncated variance of the outcome variable (reading comprehension), since it was used to divide the sample into two groups via a cutoff point.

However, this remarkably low predictive power within the struggling-reader group may also indicate that functional literacy in adulthood relies on mechanisms beyond decoding and oral comprehension. As suggested by extended SVR frameworks, unmeasured cognitive and environmental constructs account for this missing variance. On a cognitive level, broader executive function—such as working-memory capacity [19,54], perspective-taking [14], background knowledge [15], and domain-general cognition [22]—are required to navigate and integrate complex functional texts. On an environmental level, socio-educational factors likely play a critical role. For instance, the alarming Polish results of PIAAC cycle 2 were partly attributed to low respondent motivation and test-taking fatigue [5], factors neglected by the SVR, but influenced by an individual’s accumulated educational experiences. Lastly, the small amount of variance explained is a strong indicator that LRs are not a homogeneous group, a hypothesis we subsequently confirmed through our latent profile analysis.

### 4.2. Heterogeneity of the LRs

Our latent profile analysis (LPA) confirmed that adults with low functional literacy in our sample were not a homogeneous group suffering from a uniform deficit. Instead, we identified three qualitatively distinct subprofiles: a language-deficit group (53.8%), a cognitively typical group (37.5%), and a dyslexic group (8.8%).

### 4.3. The Dyslexia Profile

This group, which was the smallest (n = 7, 8.8% of LRs) showed greatest deficits in decoding, RAN, and PA&M, representing a classic dyslexia profile. Most of its members (5/7 = 71.4%) declared that they had dyslexia (formally diagnosed or not) compared to only 10%–26% in other groups, providing external validity to this label. Across background measures, the dyslexia group showed medians similar to other LRs, but the small sample of this group limited the statistical power of post hoc testing. The only measure that showed significant differences to TRs was the number of books at home at the age of 14. The majority of this group reported that there were fewer than 10 books at their childhood home compared to 26–100 books reported by the TRs (medians).

A small number of probably dyslexic individuals among our poor comprehenders may look surprising. However, severely dyslexic individuals with profound decoding difficulties are unlikely to have agreed to take part in such study, thus underestimating the true proportion of dyslexic individuals among poor adult comprehenders. Nonetheless, given this group size, this class must be treated as a provisional profile, requiring replication in larger samples.

### 4.4. Language-Deficit Profile

More than half the LRs were grouped by LPA as the language-deficit subsample. Empirically, these adults possess preserved decoding skills, but suffer from moderate-to-severe deficits in broader oral language and listening comprehension. They likely learned decoding skills fairly easily in the early school years, but showed weaknesses in vocabulary and possibly grammar. We hypothesize that in transparent orthographies, where basic decoding is often mastered within the first year of instruction [16,27], individuals with underlying lexical or grammatical weaknesses may be left behind as the curriculum shifts from “learning to read” to “reading to learn.” Without early intervention targeting oral language, the hidden deficit may compound over decades [55]. The exploratory questionnaire data aligns with this hypothesis, illustrating a pattern consistent with the Matthew effect [29], but also their current socioeconomic and digital marginalization. Questionnaire data show that they grew up in an impoverished home literacy environment (median of fewer than 10 books in their childhood home) and retrospectively reported greater difficulty when learning to read. While our cross-sectional data cannot prove causality, it is plausible that this early struggle initiated a lifelong cycle of reading avoidance, which was reflected in their current habits: language-deficit adults reported significantly lower frequencies of reading books or everyday texts in adulthood, using the internet for shopping, or keeping in touch with others, but also using math. They also reported poorer financial situations and self-assessed their reading skills as lower, all in comparison to the TR group.

### 4.5. The Cognitively Typical Profile

The cognitively typical profile (class 2; 37.5%) exhibited performances across decoding, RAN, PA&M, language, and listening comprehension comparable with TRs. Still, they failed the functional reading test qualifying them as LRs. Similar groups with typical-like performance on cognitive and reading-related tasks were found in previous studies [18]. Because our measures cannot fully explain this discrepancy, we offer two post hoc interpretations. First, functional RC, unlike the direct cognitive measures administered in our one-on-one Google Meet battery, demands skills beyond foundational reading components. PIAAC-style functional tasks require nonlinear text navigation: rapidly scanning, identifying relevant information, suppressing distractions, and integrating findings across multiple text sections. Second, we speculate that testing context may play a role. The assessment session was administered in a supervised one-on-one setting, which probably improved focus, whereas the reading comprehension screening was unsupervised and low-stakes. It is possible that a lack of test-taking motivation in the unsupervised environment depressed the scores, a phenomenon previously hypothesized to affect Polish PIAAC results [5]. But motivation to read is not separate from reading skill per se: they are likely to be reciprocally related. Questionnaire data provide additional information: the cognitively typical participants, although matching the TR group across the measured composites, had significantly impoverished childhood literacy environments, did not read as many texts in everyday life, and did not use the internet to keep in touch with the others as much as the TR group. Conversely, they retrospectively reported much less struggle when learning to spell in primary school when compared to the language-deficit class.

### 4.6. Practical Implications for Education and Everyday Literacy

The heterogeneity uncovered by the LPA carries implications for adult-literacy policies and instruction methods. Treating all struggling readers as if they have decoding deficits will not work: it will not help the language-deficit group who struggle with vocabulary. Furthermore, as evidenced by the cognitively typical profile, functional literacy captures something more complex than the sum of its parts. Attempts to support struggling adult readers must recognize the role of engagement and motivation, and try to foster learners’ sense of competence and relatedness [56,57].

These distinct profiles also highlight gaps in earlier stages of reading instruction. In transparent orthographies like Polish, basic decoding accuracy is typically achieved early in primary school. However, the language-deficit profile in our sample suggests a failure to adequately monitor and develop vocabulary and listening comprehension in later educational stages.

Finally, our findings underscore the everyday literacy challenges faced by this population. When navigating a medical leaflet, a tax form, or bureaucratic instructions, the barrier for the language-deficit group is not decoding the words, but integrating low-frequency vocabulary and dense syntax into a coherent mental model. Conversely, for the dyslexic profile, the sheer cognitive effort required to read a multipage document will likely lead to task abandonment. Therefore, while tailored adult basic education remains essential, the most immediate societal intervention must be environmental: institutions should adopt communication standards to align public texts with profiles of struggling readers.

### 4.7. Online Assessment Validity

Use of mobile devices imposed a penalty on participants’ comprehension score of approximately 2.5 points in both groups, and mobile devices were preferred by the LRs. While the structural models demonstrated that deficits in decoding and listening comprehension remain the primary constraints on reading even after controlling for device type, the modality effect itself warrants consideration.

Firstly, the penalty associated with mobile testing may reflect interface constraints. Functional reading tasks required navigating nonlinear texts and scanning for information. Performing these actions on a smartphone screen forced the reader to scroll between the text and the questions, inflating the working-memory load [58,59].

However, because participants choose their testing devices, alternative explanations must also be considered. First, device choice was confounded by the testing environment. Desktop use often occurs in structured, quiet settings. Mobile use is portable, leaving participants more susceptible to distractions [60]. Second, reliance on smartphones may act as a proxy for the “mobile underclass” phenomenon [61], where adults from lower socioeconomic backgrounds, with lower digital literacy skills depend on mobile devices as their primary or sole means of internet access.

These findings highlight a challenge for adult-literacy research. Studies show that lower-skilled adults often allocate less time to digital items and exhibit distinct, less engaged time-allocation profiles, suggesting that performance on computer-based literacy measures reflects not only underlying reading skill but also how adults interact with digital assessments [62]. Future digital assessments and distance-learning programs must account for this modality effect to avoid systematically underestimating the true literacy abilities of disadvantaged populations.

### 4.8. Limitations

The sample in our study should not be interpreted as representative of the adult population or struggling readers in Poland. By design, it excluded tertiary-educated adults and relied on self-selection, which likely underrepresented the lowest performers in reading and favored participants with basic IT skills, able and willing to engage with a reading-focused study. Ironically, individuals with higher education were the most eager to participate. While our financial compensation was generous, an adult whose literacy skills are so low that they cannot navigate an online advertisement or read an initial consent form is excluded by the very design of the study.

Secondly, the employment of an extreme-group design alters sample variance. While this design maximizes discriminative power between groups, it truncates the middle of the distribution, restricting the variance within each subgroup. Consequently, the low proportion of variance explained in our multigroup path models—particularly the R^2^ = 0.045 in the LR group—is probably an artifact of this range restriction rather than solely reflecting unmeasured constructs. Conversely, the exclusion of intermediate readers (26–30 points) upon recruitment likely inflated the between-group effect sizes and statistical significance of predictors like decoding and listening comprehension in our initial classification model. The structural parameters should therefore be interpreted as pathways that distinguish the extremes of reading proficiency, rather than continuous developmental trajectories generalizable to the broader public.

Additionally, while the latent profile analysis provides statistical support for the existence of distinct subgroups among struggling readers, the small sample of the dyslexia profile necessitates treating this specific class as provisional until replicated in larger, independent samples. Due to sample constraints, we were unable to cross-validate these profiles in an independent cohort. Furthermore, our subsequent interpretations regarding the developmental trajectories (e.g., the Matthew effect) and contextual drivers (e.g., test-taking motivation) of these profiles are largely speculative. Longitudinal designs and experimental manipulations are required to directly test these explanatory hypotheses.

Our study design involved necessary methodological and statistical trade-offs. To prevent participant fatigue, our behavioral battery was designed to last a maximum of 1 h. This required omitting measures of certain critical components—most notably working memory and morphological awareness—which limited our ability to test extensions of the SVR models. The substantial unexplained variance in the low-reader group suggests that unmeasured factors play a critical role, and future research with larger samples should integrate them into extended structural models. However, given the scarcity of adult data in transparent orthographies, establishing the validity of the classic SVR model was a necessary first step, leaving the integration of extended predictors (such as background knowledge and working memory) to future research.

Furthermore, lacking adult-normed cognitive assessments in Poland, to some extent we relied on in-house measures and child-adapted tasks. As noted in the Introduction, how reading components are operationalized shapes the resulting structural pathways and typologies. Therefore, the results must be interpreted cautiously, as child-adapted tasks may fail to fully capture adult cognitive complexity. Additionally, sample-size constraints necessitated using unit-weighted manifest composites rather than full latent structural equation modeling, failing to partial out measurement error. This error generally attenuates regression estimates, suggesting the true associations between our predictors and reading comprehension may be stronger than observed [63].

## 5. Conclusions

This study suggests that functional reading difficulties in Polish adults are not homogeneous, but rather a result of diverse factors. While the transparency of the Polish writing system may allow most adults to achieve basic accuracy, struggling readers often fail to reach the level of fluency and automation necessary for efficient comprehension. Specifically, the research identified three distinct profiles among low readers in our sample: language deficit, cognitively typical, and dyslexic. It shows that interventions must be tailored to their specific needs. These findings underscore the need for specialized adult-literacy support in Poland to address the growing challenge of low functional literacy and its associated social and economic risks.

## Figures and Tables

**Table 1:** Demographic characteristics of typical readers and low readers.

Variable	Total (N = 158)	Typical Readers (n = 76)	Low Readers (n = 82)	Test Statistic	*p*
**Age,** ***M*** **(** ***SD*** **)**	41.03 (13.16)	40.24 (11.91)	41.76 (14.25)	*t*(154.4) = −0.73	0.467
**Sex**				χ^2^(1) = 1.10	0.295
Female	93 (58.9%)	41 (53.9%)	52 (63.4%)		
Male	65 (41.1%)	35 (46.1%)	30 (36.6%)		
**Residence**				χ^2^(3) = 5.49	0.139
Village	83 (52.5%)	36 (47.4%)	47 (57.3%)		
Town (<20,000)	38 (24.1%)	16 (21.1%)	22 (26.8%)		
Small city (20–100,000)	15 (9.5%)	10 (13.2%)	5 (6.1%)		
Large city (>100,000)	22 (13.9%)	14 (18.4%)	8 (9.8%)		
**Education**				χ^2^(3) = 13.63	0.001
Primary (*podstawowe*) and lower secondary (*gimnazjum*, defunct)	26 (16.5%)	10 (13.2%)	16 (19.5%)		
Basic vocational (*zawodowe*)	89 (56.3%)	35 (46.1%)	54 (65.9%)		
General secondary (*liceum*) and technical secondary (*technikum*)	43 (27.2%)	31 (40.8%)	12 (14.6%)		

Note: Group assignment based on Czytest score.

**Table 2:** Descriptive statistics and group comparisons for decoding and listening comprehension.

	Low Readers *M* (*SD*)	Typical Readers *M* (*SD*)	*t*	df	*p*	Cohen’s d [95% CI]
Decoding	−0.27 (0.99)	0.29 (0.46)	4.61	115.91	<0.001	0.72 [0.39, 1.04]
Listening Comprehension	−0.34 (0.81)	0.37 (0.73)	5.87	155.89	<0.001	0.93 [0.60, 1.26]

**Table 3:** Group differences on ordinal variables after FDR correction medians [Q1–Q3] and Dunn’s post hoc test results across reading profiles.

	Reading Profiles (Medians [Q1–Q3])				Omnibus Test	
Variable	TRs	LRs C1: Language	LRs C2: Cognitively Typical	LRs C3: Dyslexic	H	*p*
Books at Home (Age 14)	2 [1–2] ^a^	0 [0–1] ^b^	0 [0–2] ^b^	0 [0–0] ^b^	28.72	<0.001
Internet for Contact Use	4 [3–4] ^a^	3 [1–4] ^b^	3 [2–4] ^b^	4 [0–4] ^ab^	25.51	<0.001
Frequency: Reading Texts	4 [3–4] ^a^	3 [1–4] ^b^	3 [1–4] ^b^	2 [2–4] ^ab^	17.44	0.004
Financial Situation	2 [2–2] ^a^	2 [1–2] ^b^	2 [1–2] ^ab^	1 [1–2] ^ab^	14.46	0.010
ARHQ: Learning to Spell	1 [0–2] ^ab^	2 [1–3] ^a^	1 [0–1] ^b^	2 [2–2] ^ab^	14.22	0.010
ARHQ: Learning to Read	0 [0–1] ^a^	2 [1–2] ^b^	1 [0–2] ^ab^	2 [1–2] ^ab^	13.72	0.011
Online Shopping Frequency	3 [2–4] ^a^	1 [0–4] ^b^	2 [1–3] ^ab^	3 [0–4] ^ab^	11.42	0.026
Math Use	3 [2–3] ^a^	1 [1–3] ^b^	2 [1–3] ^ab^	2 [2–3] ^ab^	10.17	0.036
Self-Assessed Reading Skill	3 [3–4] ^a^	3 [2–3] ^b^	3 [3–4] ^ab^	2 [2–3] ^ab^	10.45	0.036
Frequency: Reading Books	2 [1–3] ^a^	1 [0–2] ^b^	1 [0–3] ^ab^	1 [1–2] ^ab^	9.47	0.045

Notes: Values represent medians [Q1–Q3]. H = Kruskal–Wallis statistic. Omnibus *p*-values were corrected using Benjamini–Hochberg FDR. Groups sharing the same superscript letter do not differ significantly (*p* > 0.05) based on Dunn’s post hoc tests with Holm correction.

## Data Availability

The original data presented in the study are openly available in OSF at https://osf.io/hf86a/ accessed on 16 April 2026.

## Supplementary Materials

The following supporting information can be downloaded at https://www.mdpi.com/article/10.3390/brainsci16050438/s1 Supplement Section S1. Modality Effects in Online Reading Assessment. Supplement Section S2. Ordinal Variables Tested in Research Question 4. Supplement Section S3. Participant Questionnaire Items, Variable Codes, and Response Options.

## Abbreviations

The following abbreviations are used in this manuscript.

TRs	typical readers
LRs	low readers
RC	reading comprehension
SVR	Simple View of Reading
FDR	false-discovery rate
LPA	latent path analysis
RAN	rapid automatized naming
PA&M	phonological awareness and memory
RQ	research question
