# Supplementary material for: Cognitive Underpinnings of Functional Reading Difficulties in Polish Adults
Source: Brain Sci. 2026 Apr 22;16(5):438. doi: 10.3390/brainsci16050438 (PMC13204182; doi:10.3390/brainsci16050438)
Supplement: Supplementary file 1 [file brainsci-16-00438-s001.zip › brainsci-4254823-supplementary (2).pdf]

Supplementary Materials

Supplement Section S1

The Modality Effect in Online Literacy Assessment

The present study employed a remote, self-administered testing protocol for the initial functional reading comprehension screening (*Czytest*). Because participants could complete the assessment on their own devices, we investigated whether the testing modality (Mobile vs. Computer) systematically affected reading scores and group classification.

1. Descriptive Statistics and Device Distribution

Participants were categorized into two device groups based on self-report: Mobile (smartphones, tablets; n = 70) and Computer (desktop, laptop; n = 88). A Chi-square test of independence revealed a significant association between reading proficiency group and device choice, chi2(1, N = 158) = 12.82, p < .001. As illustrated in Figure S1, the majority of Typical Readers (71.1%) completed the assessment on a computer, whereas the majority of Low Readers (58.5%) relied on a mobile device.

Figure S1. Percentage of participants using mobile versus computer devices by reading proficiency group.

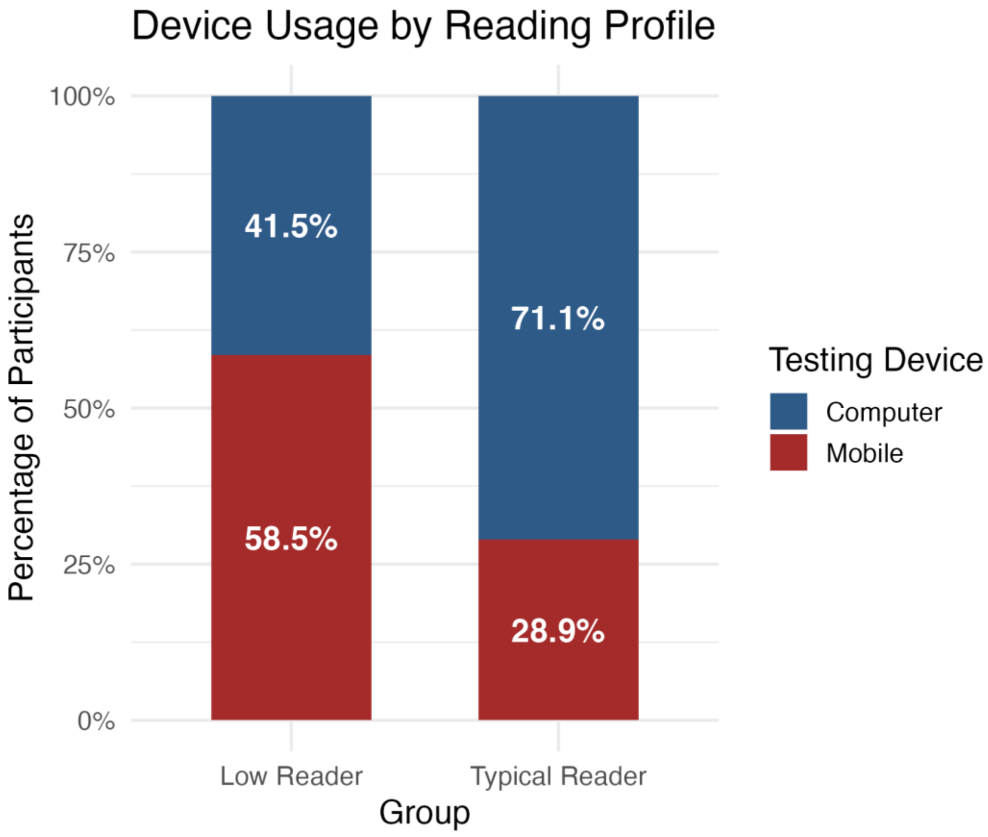

2. The Modality Penalty on Reading Comprehension

To determine if device type systematically influenced test outcomes, we conducted a 2x2 Analysis of Variance (ANOVA) predicting standardized reading comprehension scores based on Group (Typical vs. Low) and Device Type (Mobile vs. Computer).

The ANOVA revealed a massive main effect of Group ($F(1, 154) = 695.41$, $p < .001$) and a highly significant main effect of Device Type ($F(1, 154) = 12.08$, $p < .001$). There was no significant interaction ($F(1, 154) = 1.18$, $p = .280$), indicating that the penalty for using a mobile device was relatively uniform across both proficiency groups. Taking the test on a mobile device was associated with a systematic drop in reading comprehension scores (approximately -2.5 points on the raw Czytest scale (max 41 points).

Figure S2. Standardized reading comprehension scores by device type and reading proficiency group.

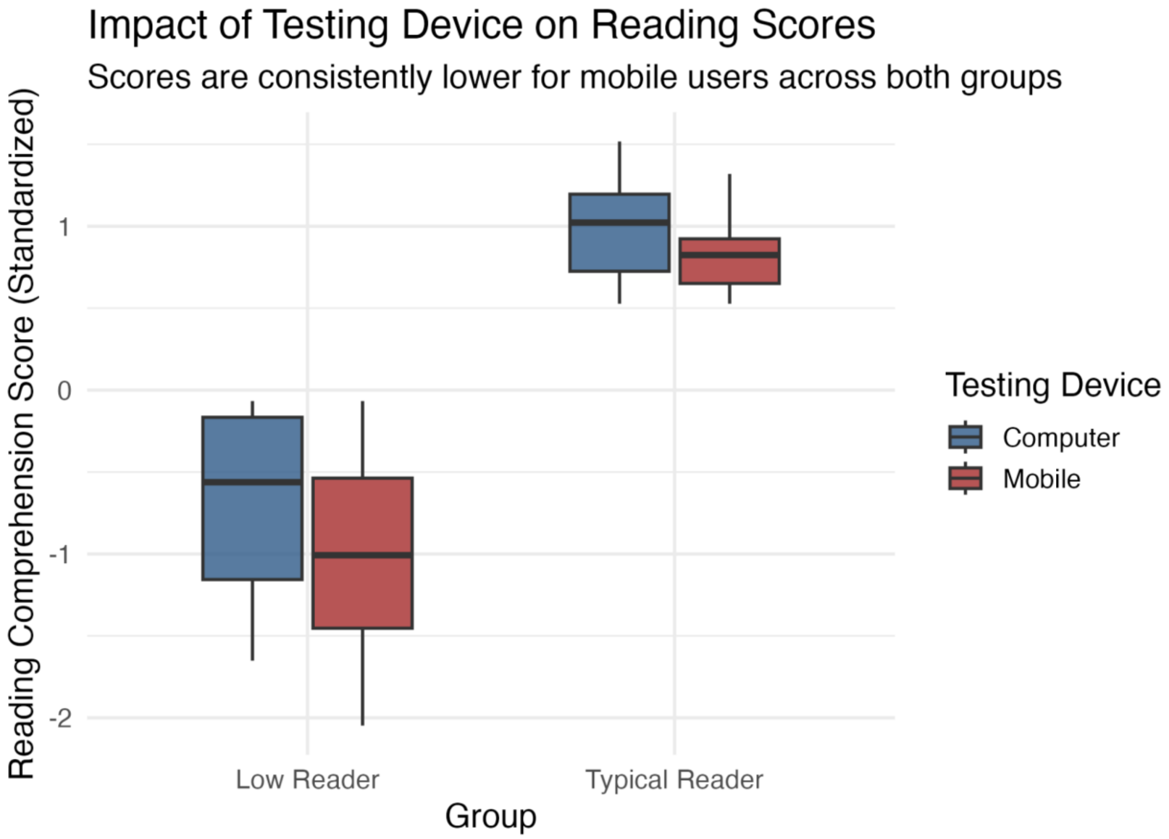

3. Impact on Structural Modeling

To ensure that our core cognitive findings were not artifacts of testing modality, we re-ran both structural models with Device Type (0 = Computer, 1 = Mobile) included as a dummy-coded covariate.

A. Updated DWLS Model

The DWLS model was specified using the RAM representation to define observed composites as single-indicator latent variables, allowing for the simultaneous estimation of continuous cognitive predictors and an ordered categorical outcome (Group_bin: 0 = Low Reader, 1 = Typical Reader).

The model demonstrated that the testing modality had a significant, independent negative effect on the probability of being classified as a TR (beta = -.367, SE = .208, $p < .001$). However, the inclusion of this covariate did not negate the core cognitive hypothesis: both Decoding (beta = .369, SE = .140, $p < .001$) and Listening Comprehension (beta = .341, SE = .110, $p < .001$) remained robust, highly significant, independent predictors of functional reading difficulties.

B. Updated Multigroup Path Analysis

The multigroup path model was re-estimated using Maximum Likelihood with robust standard errors (MLR) to predict the standardized Czytest reading comprehension scores within each proficiency group while controlling for Device Type. The updated model exhibited excellent fit to the data (chi2(20) = 19.55, p = 0.486; CFI = 1.000; TLI = 1.002; RMSEA = 0.000; SRMR = 0.036).

Controlling for testing modality did not alter the primary structural mechanisms identified in the main manuscript. In TR Listening Comprehension remained the sole significant cognitive predictor of reading scores (beta = .325, SE = .044, p = .005), while Decoding was non-significant (beta = .115, p = .392). In LR, Decoding remained the primary cognitive constraint (beta = .242, SE = .042, p = .001), while Listening Comprehension was non-significant (beta = -.085, p = .489).

However, the Device Type covariate itself emerged as a significant negative predictor across both groups. Taking the test on a mobile device significantly depressed reading comprehension scores for Typical Readers (beta = -.260, SE = .058, p = .007) and Low Readers (beta = -.296, SE = .113, p = .003). Notably, the inclusion of this contextual covariate increased the proportion of explained variance (R2) in reading comprehension scores from 16.0% to 22.1% in the Typical Reader group, and from 4.5% to 14.0% in the Low Reader group.

These results confirm that while online functional reading assessments may inadvertently conflate cognitive capacity with hardware constraints - a finding with significant implications for remote testing validity - the distinct cognitive mechanisms driving reading proficiency in transparent orthographies remain structurally robust.

Supplement Section S2

S2 presents all ordinal variables tested in the fourth research question. Significant results along with post-hoc tests are presented in Table 3 in the main text.

Table S1

**Variable**	**H_stat**	**p_raw**	**p_fdr**
ARHQ: Learning to Read	13.72	0.003	0.011
arh_0_ARHQ_learnreading			
ARHQ: Learning to Spell	14.22	0.003	0.010
arh_2_ARHQ_learnspelling			
ARHQ: Reading Skill	7.49	0.058	0.084

arh_1_ARHQ_readingskill			
Books at Home (Age 14)	28.72	< .001	< .001
arh_3_booksat14			
Financial Situation	14.46	0.002	0.010
met_11_finances			
Financial Situation Worry	7.44	0.059	0.087
met_12_finances_worry			
Frequency: Reading Books	9.47	0.024	0.045
arh_4_readbooks			
Frequency: Reading Texts	17.44	< .001	0.004
arh_5_readtexts			
Locus of Control	3.60	0.309	0.391
arh_11_locuscontrol			
Math Use	10.17	0.017	0.036
met_16_maths			
Mother's Education	8.19	0.042	0.073
met_13_motheredu			
Online Shopping Frequency	11.42	0.010	0.026
met_14_wwwshopping			
Reading Help Received	7.62	0.055	0.084

arh_7_readhelp			
Self-Assessed Health *met_17_health*	1.14	0.767	0.767
Self-Assessed Reading Skill *arh_6_readassed*	10.45	0.015	0.036
Sight Quality *met_19_sight*	2.56	0.464	0.551
Sleep This Month *met_21_sleepmonth*	3.85	0.278	0.377
Sleep Today *met_20_sleeptoday*	2.32	0.509	0.569
Internet For Contact Use *met_15_wwwsocial*	25.51	< .001	< .001
Social Trust *arh_10_socialtrust*	2.03	0.566	0.597

Note. H = Kruskal-Wallis statistic. p_raw = uncorrected p-value. p_fdr = p-value corrected using Benjamini-Hochberg FDR across all ordinal variables.

Supplement Section S3

S3 presents all questions asked to participants, their codes, and response options.

met_0_sex: Please indicate your gender.

1. Woman
2. Man
3. Other

met_1_year: In which year were you born? (Open response)

met_2_lang: Is Polish your native language?
1. Yes
2. No

met_3_langother: What is your native language? (Open response)

met_4_home: In what type of settlement do you live?
1. Village
2. Small town (under 20,000 residents)
3. Medium town (20,000 - 100,000 residents)
4. Large town (over 100,000 residents)

met_5_education: What is your highest completed level of education?
1. Primary
2. Lower secondary
3. Vocational
4. General secondary (high school)
5. Vocational secondary (technical school) or post-secondary
6. Tertiary

met_6_student: Are you currently studying or attending school to obtain a certificate or diploma?
1. Yes
2. No

met_7_employment: What best describes your current employment situation?
1. Employed for pay
2. Looking for work
3. Studying or in school
4. Retired or on disability pension
5. Caring for home, child, or family

met_8_job_employed: What is your occupation? (Open response)

met_9_studies_what: Where are you studying or attending school? (Open response)

met_10_job_retirement: What was your occupation before retirement or disability? (Open response)

met_11_finances: Which of the following best characterizes how money is managed in your household?
1. I live very poorly - not enough even for basic needs
2. I live modestly - we have to manage very frugally day-to-day
3. I live averagely - enough for daily needs, but we save for major purchases
4. I live well - enough for many things without special saving

5. I live very well - I can afford some luxury

met_12_finances_worry: Which of the following statements would you choose as most consistent with your household's current financial situation?

1. I am confident that I will manage financially.
2. I am not afraid of poverty, though I worry my material situation may worsen.
3. I fear poverty, but think we will manage somehow.
4. I fear poverty and do not know how I will manage.

met_13_motheredu: What was the highest level of education completed by your mother or legal guardian?

1. Primary
2. Vocational
3. Secondary or post-secondary
4. Tertiary
5. Don't know / don't remember

met_14_wwwshopping: How often do you use a computer, tablet, or phone for online banking or shopping?

1. Never
2. Less than once a month
3. Less than once a week, but at least once a month
4. At least once a week, but not daily
5. Daily

met_15_wwwsocial: How often do you use a computer, tablet, or phone to communicate with others, e.g., via email, social media, or voice calls over the Internet?

1. Never
2. Less than once a month
3. Less than once a week, but at least once a month
4. At least once a week, but not daily
5. Daily

met_16_maths: How often do you use simple calculations or formulas?
Never

1. Less than once a month
2. Less than once a week, but at least once a month
3. At least once a week, but not daily
4. Daily

met_17_health: How would you describe your health status?

1. Very good
2. Good
3. Average
4. Poor

5. Very poor

met_18_badhealth: Do health problems limit your ability to perform certain activities?
1. Yes, severely limited
2. Yes, limited but not severely
3. No, no limitations
4. Hard to say

met_19_sight: How would you describe your eyesight (with glasses or contacts if worn)?
Very good
1. Good
2. Average
3. Poor
4. Very poor

met_20_sleeptoday: How many hours did you sleep last night?
1. 4 hours or less
2. 5 hours
3. 6 hours
4. 7 hours
5. 8 hours or more

met_21_sleepmonth: How many hours do you usually sleep per day over the last month?
1. 4 hours or less
2. 5 hours
3. 6 hours
4. 7 hours
5. 8 hours or more

met_22_workphysic: How often does your work involve prolonged physical labor?
1. Never
2. Less than once a month
3. Less than once a week, but at least once a month
4. At least once a week, but not daily
5. Daily

met_23_workroutine: To what extent can you choose or change the order of tasks in your work?
1. Not at all
2. To a very small extent
3. To some extent
4. To a large extent
5. To a very large extent

met_24_workreading: How often do you read in your work? (For example: instructions, emails, work notes, articles, books.)

1. Never
2. Less than once a month
3. Less than once a week, but at least once a month
4. At least once a week, but not daily
5. Daily

arh_0_ARHQ_learnreading: How much difficulty did learning to read cause you in primary school?
1. None
2. Slight
3. Average
4. Significant
5. Very significant

arh_1_ARHQ_readingskill: How would you rate your reading skill in primary school compared to other children?
1. Very good
2. Good
3. Average
4. Poor
5. Very poor

arh_2_ARHQ_learnspelling: Did you have trouble learning spelling in primary school?
1. None
2. Slight
3. Average
4. Significant
5. Very significant

arh_3_booksat14: Approximately how many books were in your home when you were 14? (Do not include magazines, newspapers, or school textbooks. One meter of shelving is about 40 books.)
1. 10 books or fewer
2. 11 to 25 books
3. 26 to 100 books
4. 101 to 200 books
5. 201 to 500 books
6. Over 500 books

arh_4_readbooks: How often do you read books? (Include both paper and electronic books.)
1. Never
2. Less than once a month
3. Less than once a week, but at least once a month
4. At least once a week, but not daily
5. Daily

arh_5_readtexts: How often do you read other texts? (For example: newspapers, news, or practical information on the Internet.)

1. Never
2. Less than once a month
3. Less than once a week, but at least once a month
4. At least once a week, but not daily
5. Daily

arh_6_readassed: How would you generally rate your reading skill?

1. Very poor
2. Poor
3. Average
4. Good
5. Very good

arh_7_readhelp: How often do you need help reading instructions, leaflets, or other materials from a doctor or pharmacy?

1. Always
2. Often
3. Sometimes
4. Rarely
5. Never
6. I do not read such materials

arh_8_dyslexia: Do you have a diagnosis of dyslexia? (Dyslexia is serious difficulty learning to read and reading.)

1. Yes, I have a dyslexia diagnosis
2. Yes, I have a dyslexia diagnosis, but my problems were mainly with writing
3. I think I have dyslexia, though I have no diagnosis
4. I do not think I have dyslexia

arh_9_fhd: Did anyone in your close family have serious difficulties with reading and writing?

1. Yes
2. No

arh_10_socialtrust: To what extent do you agree with the statement: "If you are not careful, other people will take advantage of you."

1. Strongly disagree
2. Somewhat disagree
3. No opinion
4. Somewhat agree
5. Strongly agree

arh_11_locuscontrol: To what extent do you agree with the statement: "People like me have no say in what the government does."

1. Strongly disagree
2. Somewhat disagree
3. No opinion
4. Somewhat agree
5. Strongly agree

oce_0_correct: There were 41 items in this test. How many do you think you solved correctly?
1. 8 items or fewer (just a few)
2. 9-16 items
3. 17-24 items (about half)
4. 25-32 items
5. 33 items or more (almost all)

oce_1_focus: How focused were you reading texts and solving items?
1. Not at all focused
2. Not very focused
3. Somewhat focused
4. Very focused

oce_2_effort: How much effort did you put in reading texts and solving items?
1. No effort at all
2. Not much effort
3. Some effort
4. A lot of effort

oce_3_hurry: Were you in a hurry when taking this test?
1. Not in a hurry at all
2. Not really in a hurry
3. Somewhat in a hurry
4. Very much in a hurry

oce_4_device: What type of device are you using to take the test?
1. Smartphone
2. Tablet
3. Laptop/notebook
4. Desktop computer

oce_5_coursor: How are you moving the cursor right now when answering this question?
1. Finger on touchscreen
2. Mouse connected to computer
3. Touchpad

oce_6_comment: If you have any comments about this part of the test, please share them. (Open response)
